# Rhombohedral Boron Monosulfide as a p-Type Semiconductor

**DOI:** 10.3390/molecules28041896

**Published:** 2023-02-16

**Authors:** Norinobu Watanabe, Keisuke Miyazaki, Masayuki Toyoda, Kotaro Takeyasu, Naohito Tsujii, Haruki Kusaka, Akiyasu Yamamoto, Susumu Saito, Masashi Miyakawa, Takashi Taniguchi, Takashi Aizawa, Takao Mori, Masahiro Miyauchi, Takahiro Kondo

**Affiliations:** 1 Graduate School of Pure and Applied Sciences, University of Tsukuba, Tsukuba 305-8573, Japan; 2 Department of Materials Science and Engineering, School of Materials and Chemical Technology, Tokyo Institute of Technology, Meguro-ku, Tokyo 152-8552, Japan; 3 Department of Physics, Tokyo Institute of Technology, Meguro-ku, Tokyo 152-8551, Japan; 4 Tsukuba Research Center for Energy Materials Science, Department of Materials Science, Faculty of Pure and Applied Sciences, University of Tsukuba, Tsukuba 305-8573, Japan; 5 R&D Center for Zero CO_2_ Emission with Functional Materials, University of Tsukuba, Tsukuba 305-8573, Japan; 6 International Center for Materials Nanoarchitectonics (WPI-MANA), National Institute for Materials Science (NIMS), 1-1 Namiki, Tsukuba, Ibaraki 305-0044, Japan; 7 Institute of Engineering, Tokyo University of Agriculture and Technology, Tokyo 183-8538, Japan; 8 Research Center for Functional Materials, National Institute for Materials Science, Tsukuba 305-0044, Japan; 9 Advanced Research Center for Quantum Physics and Nanoscience, Tokyo Institute of Technology, Meguro-ku, Tokyo 152-8551, Japan

**Keywords:** rhombohedral boron monosulfide, two-dimensional materials, seebeck coefficient

## Abstract

Two-dimensional materials have wide ranging applications in electronic devices and catalysts owing to their unique properties. Boron-based compounds, which exhibit a polymorphic nature, are an attractive choice for developing boron-based two-dimensional materials. Among them, rhombohedral boron monosulfide (r-BS) has recently attracted considerable attention owing to its unique layered structure similar to that of transition metal dichalcogenides and a layer-dependent bandgap. However, experimental evidence that clarifies the charge carrier type in the r-BS semiconductor is lacking. In this study, we synthesized r-BS and evaluated its performance as a semiconductor by measuring the Seebeck coefficient and photo-electrochemical responses. The properties unique to p-type semiconductors were observed in both measurements, indicating that the synthesized r-BS is a p-type semiconductor. Moreover, a distinct Fano resonance was observed in Fourier transform infrared absorption spectroscopy, which was ascribed to the Fano resonance between the E(2) (TO) phonon mode and electrons in the band structures of r-BS, indicating that the p-type carrier was intrinsically doped in the synthesized r-BS. These results demonstrate the potential future application prospects of r-BS.

## 1. Introduction

Two-dimensional (2D) materials exhibit unique properties such as a large surface area and unique electronic states [1,2,3]. Hence, they have potential applications in the development of superior electronic devices and catalysts. Among them, boron has a number of stable structures owing to its polymorphism [4,5,6]. Theoretical predictions indicate that borophene [6,7,8,9], boron hydride [10], boron sulfide [11], boron oxide [12], and boron phosphide [13] form stable 2D phases; hence, these structures have attracted much attention as targets for developing new 2D materials [14]. Among them, rhombohedral boron monosulfide (r-BS) has been successfully synthesized experimentally [15] and is theoretically predicted to exhibit excellent thermal conductivity [16] and high hydrogen storage performance via alkali modification [17]. Moreover, experimental reports have indicated that r-BS can be easily exfoliated from bulk to nanosheets physically in the air [18]. Furthermore, experimental results as well as density functional theory (DFT) calculations have shown that the bandgap of r-BS varies with the number of layers [18]. Hence, r-BS has the potential for a wide range of applications. By contrast, the bandgap of r-BS is always indirect and independent of the number of layers, according to the theoretical calculations [18], which is in sharp contrast to the case of intriguing 2D materials like MoS_2_, which shows a transition from indirect to direct bandgap by changing the number of layers from bulk to monolayer [1]. As for the effective mass of r-BS, the average effective electron and hole masses are reported to change from 0.41 to 0.29 and 0.57 to 6.95 by changing the number of layers from bulk to monolayer, respectively, based on the theoretical calculations [18]. This means that the mobility of the carrier in r-BS is quite different depending on the type of carrier. However, the properties of r-BS as a semiconductor have not been experimentally investigated. Semiconducting borides have predominantly exhibited p-type characteristics. In 3D-structured borides, this is due to the particular bonding of the boron clusters [19] and electron deficiency. The 2D borides are much less well known, although n-type behavior has generally been proven to be much more difficult to induce in h-BN [20]. Therefore, it is of high interest to investigate r-BS.

In this study, r-BS was synthesized and its structure and optical properties were evaluated via X-ray diffraction (XRD), Fourier transform infrared absorption spectroscopy (FT-IR), Raman scattering, and UV-visible spectroscopy. The Seebeck coefficient and photo-electrochemical responses were measured as well. These measurements revealed that the synthesized r-BS shows the properties of a p-type semiconductor driven by defects.

## 2. Results and Discussion

### 2.1. Structure of Synthesized r-BS

r-BS was synthesized based on the methods described in previous studies [15,18,21,22]. Powdered sulfur and amorphous boron—used as starting materials—were mixed at an atomic ratio of 1:1, and pellets of the samples were formed. The pellets were heated to 1873 K at a pressure of 5.5 GPa and then quenched to room temperature (approximately 300 K) to obtain the r-BS samples. The resulting r-BS sample was a pink pellet that could be easily powdered. The synthesis procedure is detailed in the Materials and Methods section.

**Figure 1:**
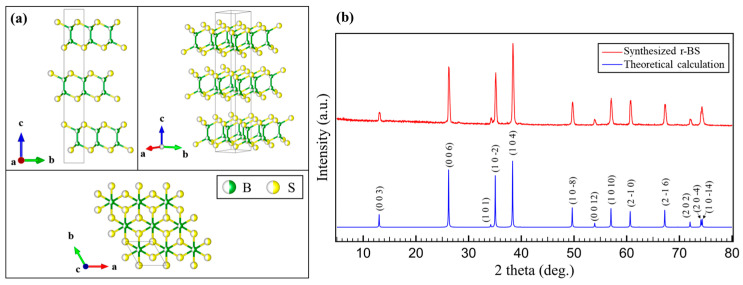
(**a**) The structure of r-BS [23] and (**b**) X-ray diffraction patterns (CuKα: *λ* = 1.5418 Å) of r-BS obtained via a high pressure synthesis method, and those of the theoretically calculated structure [18].

As shown in Figure 1a, the structure of r-BS has an R-3m symmetry. r-BS consists of boron and sulfur layers periodically stacked in an A-B-C stacking manner by van der Waals forces. This unique structure is similar to those of the transition metal dichalcogenides such as MoS_2_: the structure of MoS_2_ is an arrangement wherein one Mo atom in the MoS_2_ structure is replaced by a pair of B atoms. However, according to a previous study [18], the nature of bonding with sulfur is rather different. B and S atoms are covalently bonded in r-BS, whereas bonds between S and transition metals (such as Mo) are ionic (the transition metal is positively charged and sulfur is negatively charged) [18]. The results of XRD measurements of the r-BS synthesized in this study are shown in Figure 1b. The structure of r-BS synthesized under the conditions of this experiment is consistent with the previously reported structure [15,18,21,22].

The size of the r-BS crystallite was roughly estimated to be 30 ± 10 nm independent of the crystal orientation, using Scherrer’s formula:

(1) $$B_{hkl}=\frac{0.94\lambda }{L_{hkl}\cos\theta },$$

where *B_hkl_* is the peak half-width (rad), *λ* is the wavelength of the X-rays used for irradiation (CuKα: 1.5406 Å), *L_hkl_* is the crystallite size, and *θ* is the diffraction angle.

### 2.2. Optical Properties of r-BS

**Figure 2:**
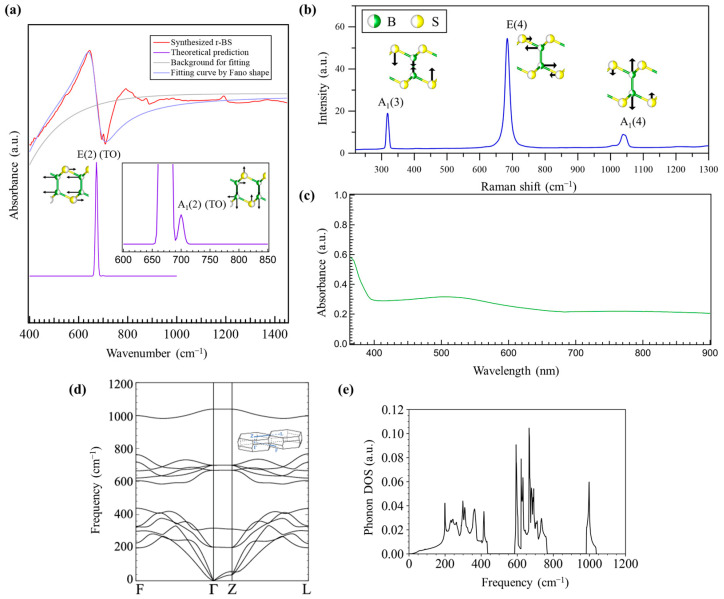
Optical properties of the synthesized r-BS powder. (**a**) Fourier transform infrared absorption spectra with fitting by Fano shapes and theoretical predictions of the E(2) (TO) and A1(2) (TO) phonon modes. (**b**) Raman scattering and (**c**) Ultraviolet-Visible absorption spectra. r-BS calculated phonon dispersion (**d**) and density of states (DOS) (**e**) of r-BS. The *k*-point path in the rhombohedral Brillouin zone is shown in the inset in panel d.

The optical properties examined in this work are shown in Figure 2. Figure 2a shows the results of FT-IR measurements of the synthesized powder, r-BS. A relatively large mountain-valley shape was observed at 673 cm^−1^, and a smaller peak was observed at the bottom of the valley at 701 cm^−1^. The smaller peak at 701 cm^−1^ was assigned to the A_1_(2) (TO) phonon mode, based on a previous study [22] and theoretical predictions [24]. Our DFT calculations also predicted the same peak, as shown by a purple curve in Figure 2a (the calculated phonon dispersion and phonon density of states are shown in Figure 2d,e, respectively). The significant smaller peak intensity is attributed to the plane-perpendicular and smaller dynamic dipole moment. The mountain-valley shape at around 673 cm^−1^ was fitted with a Fano resonance cross section *σ* as a function of energy *E* given by

(2) $$\sigma =\frac{{\left(\frac{q\Gamma_{res}}{2}+E-E_{res}\right)}^{2}}{{\left(\frac{\Gamma_{res}}{2}\right)}^{2}+{\left(E-E_{res}\right)}^{2}}$$

where *E_res_* is the resonance energy, *Γ_res_* is the resonance width, and *q* is the Fano parameter determined by the coupling between a vibrational mode and conductive electrons [25]. As shown in Figure 2a, the mountain-valley shape with the background subtraction was fitted by *E_res_* of 82.9 meV (669 cm^−1^), *Γ_res_* of 9.2 meV (74 cm^−1^), and *q* of −1.19. This means that the mountain-valley shape is derived from the Fano resonance between the E(2) (TO) phonon mode [20] and electrons in the band structures. Fano resonance has also been observed between the *E*_u_ phonon mode and conductive electrons in bilayer graphene [26,27,28]. The appearance of Fano resonance requires doping of carriers in semiconducting materials [29,30]. Therefore, herein, the Fano resonance proves that the carrier was intrinsically doped in the synthesized r-BS. In addition, a reported Fano parameter for the bilayer graphene was approximately −0.8 [27]. The slightly larger absolute value of Fano parameter for the r-BS means that the coupling of the phonon mode and electrons is more than comparable to that of bilayer graphene.

As shown in Figure 2b, the Raman scattering spectrum of the synthesized r-BS powder shows three distinct peaks at 319, 686, and 1041 cm^−1^, which are ascribed to the A1(3), E(4), and A1(4) modes of r-BS, respectively [18,22]. The absence of other peaks corresponding to boron or sulfur compounds in the Raman spectra indicates that r-BS was synthesized as a single phase.

Figure 2c shows the ultraviolet-visible adsorption spectroscopy (UV-vis) results of the synthesized r-BS powder. Two types of absorption were observed: a sharp increase at 400 nm and a gradual increase from 680 nm to 510 nm. A wavelength of 400 nm corresponds to an energy of 3.1 eV. The theoretical bandgap of bulk r-BS is approximately 2.8 eV [18]. The theoretical bandgap is usually underestimated below the experimentally determined bandgap [31,32]. Therefore, the present bandgap of r-BS is consistent with the previously reported theoretical value. The gradual increase in the absorption at 680 nm (corresponding energy is 1.8 eV) is considered to be the transition from the valence band to the localized state in the bandgap, possibly due to the presence of defects because of the p-type nature of the r-BS carrier, as discussed below. The UV-Vis spectrum of r-BS was reported by Sasaki et al., who also observed two steps of optical absorption [15]. The strong absorption in the UV region was assigned to bandgap excitation, while the weak absorption in the visible light range was ascribed to defects in r-BS. The presence of defects in r-BS is indicated by the electron spin resonance (Figure S1 from Supplementary Materials), i.e., the observed distinct peaks can be ascribed to the unpaired electrons due to the presence of defects in r-BS, such as sulfur and/or boron vacancies and/or impurities. Previously reported ESR signals with g = 2.0037 for r-BS were consistently attributed to r-BS electric carriers originating from deficiency [15].

### 2.3. p-Type Property of Synthesized r-BS

**Figure 3:**
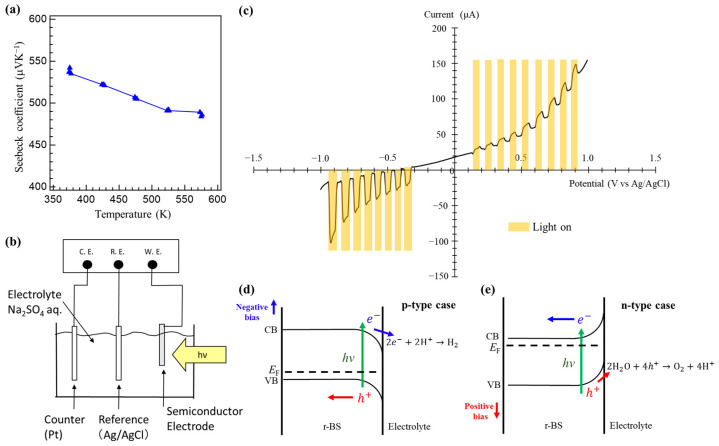
p-type property of the synthesized r-BS. (**a**) Temperature dependence of the Seebeck coefficient of the synthesized r-BS. (**b**) The experimental system used for the photoelectrochemical measurements. (**c**) Photoelectrochemical measurements of r-BS. Linear sweep voltammetry (LSV) was conducted with and without light irradiation. Schematic expected energy diagrams and photoelectrochemical reactions in the case that (**d**) r-BS is a p-type semiconductor and the case that (**e**) r-BS is an n-type semiconductor.

To identify the semiconductor type of the synthesized r-BS, the Seebeck coefficient was measured. First, an r-BS pellet was placed with flattened edges between the electrodes and chromel-alumel thermocouples on its side. A temperature difference was applied across the sample using a heater while passing a current through it, and the voltage was monitored. Figure 3a shows the Seebeck coefficient of r-BS obtained in this measurement (the measurement method is detailed in the Materials and Methods section). The measured Seebeck coefficient was in the range of 500–520 μVK^−1^. This indicates that holes are the charge carriers in the synthesized r-BS, and hence it is a p-type semiconductor. The sample has a high resistance (several MΩ) and the contact between the sample pellet and electrode may cause an error of approximately 20%. Hence, the Seebeck coefficient measurement result is merely a qualitative indication that the synthesized r-BS is a p-type semiconductor. The decrease in the Seebeck coefficient with an increase in temperature is attributed to the increase in carrier concentration. XRD measurements and FT-IR spectroscopy of the r-BS were conducted thereafter (Figures S2 and S3 from Supplementary Materials). The results show that there were no significant changes in the structure of r-BS caused by the heating during the Seebeck coefficient measurements. The sample was heated twice during the Seebeck coefficient measurements: the first round of heating included the desorption of adsorbed water (Figures S4–S6 from Supplementary Materials); the results shown in Figure 3a correspond to the Seebeck coefficient measured during the second round of heating.

Photo-electrochemical measurements were conducted to further examine the semiconductor property of r-BS. A schematic of the setup used for the measurement is shown in Figure 3b. The electrolyte was 0.5 M Na_2_SO_4_ (pH ≈ 7). The r-BS film coated on a fluorine-doped tin oxide (FTO) glass was used as a working electrode. Pt was used as the counter electrode, and Ag/AgCl was used as the reference. As shown in Figure 3c, in the dark condition, a p-type rectification profile was observed in the linear sweep voltammetry (LSV). The current was larger for the positive bias condition because the holes contributed to the current, while the photo-electrochemical current was more pronounced on the cathodic (negative bias) side. Figure 3d,e show the schematic expected energy diagrams and photo-electrochemical reactions for p-type and n-type semiconductors, respectively. For the p-type, the photo-electrochemical current is dominant when a negative bias is applied because of the hydrogen production driven by photogenerated electrons in the conduction band [33] (Figure 3d). On the other hand, an n-type semiconductor shows photocurrent at positive bias because of a water oxidation reaction, similar to the TiO_2_ and/or WO_3_ cases [34] (Figure 3e). Thus, the result in Figure 3c clearly indicates that the synthesized r-BS has a p-type nature, with holes being the majority carriers under dark conditions.

Electron spin resonance (ESR) of the r-BS before and after heating at 573 K (Figure S1 from Supplementary Materials) was performed to examine the defect state of the sample. The results indicate that finite defects exist intrinsically in the synthesized r-BS, and the amount of defect does not considerably change after heating at 573 K. These intrinsic defects may be the origin of the p-type carrier in the synthesized r-BS. Further studies are required to clarify the origin of the p-type carrier of r-BS. 

## 3. Materials and Methods

### 3.1. Starting Material

Amorphous boron (>99.5%) was prepared by the decomposition of B_2_H_6_ (Primary Metal Chemical Japan, Kanagawa, Japan). Sulfur (99%) was purchased from Wako Pure Chemical Industries Ltd., Osaka, Japan.

### 3.2. Synthesis of r-BS

Boron and sulfur were mixed at an atomic ratio of 1:1 in a mortar and then pressed at 200 kgfcm^−2^ to form pellets. The pellets were packed in h-BN capsules sandwiched between NaCl discs; they were then sandwiched between graphite discs to prepare the cell. The cells were heated at 1873 K for 40 min at a pressure of 5.5 GPa using a belt-type high-pressure apparatus with a cylinder bore diameter of approximately 32 mm [35]. The cell was then quenched and removed; the cell was crushed and separated from the r-BS sample. The sample was pink in color and could be easily crushed to obtain the powdered form.

### 3.3. X-ray Diffraction (XRD)

XRD patterns were acquired at room temperature (approximately 300 K) using a MiniFlex600 (Rigaku, Tokyo, Japan) with CuKα radiation. X-rays were generated using the line-focus principle. A reflection-free Si plate was used as the sample stage. Diffraction patterns were recorded using a D/teX Ultra silicon strip detector (Rigaku) at 0.1° s^−1^ up to a 2θ value of 80°.

### 3.4. Fourire Transform Infrared Absorption Spectroscopy (FT-IR)

FT-IR measurements were performed at room temperature using a benchtop IR equipment (ALPHAII, Bruker, Billerica, MA, USA).

### 3.5. Ultraviolet-Visible Adsorption Spectroscopy (UV-vis)

The UV-Vis absorption spectra of the r-BS powders were measured using spectrophotometer (V-750, Jasco, Tokyo, Japan) with an integration sphere unit at room temperature.

### 3.6. Seebeck Coefficient Measurement

The Seebeck coefficient values of r-BS were obtained using a ZEM-2 (ADVANCE RIKO, Yokohama, Japan). The edges of the r-BS pellet were flattened via sanding. Since the r-BS pellets alone could easily be broken up by a slight shock, pyrophyllite, which is not conductive, was left around the pellet. A carbon sheet was laid on the electrodes, with the pellets sandwiched between them. Chromel-alumel thermocouples were applied from the side. The Seebeck coefficient was measured at 373 K, 423 K, 473 K, 523 K, and 573 K. The temperature difference ΔT was applied to the sample in each temperature condition. The measurements were performed three times with different Δ*T* (1, 2, and 3 K) at each temperature condition.

### 3.7. Photoelectrochemical Measurements

The r-BS electrode was prepared on a fluorine-doped tin oxide (FTO)-coated glass by a drop casting method using r-BS powder dispersed in ethanol with a 5% NafionTM solution (DE520 CS type, Wako Pure Chemical Industries Ltd., Osaka, Japan). The photocurrent of r-BS was evaluated in an aqueous solution with 0.5 M sodium sulfate (Wako Pure Chemical Industries Ltd., Osaka, Japan) at a pH of 7.0. The working, counter, and reference electrodes were r-BS, Pt plates and Ag/AgCl, respectively. The chopping light was irradiated on the r-BS electrode using a 150 W xenon lamp, and its photocurrent was recorded by a potentiostat (Hokuto Denko Corp., HZ-7000, Tokyo, Japan) during the linear sweep voltammogram (LSV).

### 3.8. Thermogravimetry Analysis (TGA)

The experiment was performed using an STA 2500 Regulus (Netzsch Japan, Japan). The sample was placed on an Al_2_O_3_ holder, and the experiments were conducted by heating at 10 Ks^−1^ under an Ar flow.

### 3.9. ESR

ESR measurements were performed using an EMX nano (Bruker, USA). The r-BS powder was placed in a quartz tube and cooled by liquid nitrogen.

### 3.10. DFT Calculation and Simulation of Vibrational Spectrum

Density-functional theory (DFT) calculations were performed using the Quantum ESPRESSO program package [36,37]. The local density approximation was used for the exchange-correlation energy function. The norm-conserving (for electronic structures) and ultrasoft (for phonon dispersion) pseudopotentials [38,39] were used for the core electrons. The valence (B-2s, B-2p, S-3s, and S-3p) wavefunctions were expanded on a plane-wave basis with a kinetic cutoff energy of 120 Ry. The structural optimization was performed until the remaining atomic forces fell below 10^−4^ a.u. In order to calculate the vibrational properties, the density-functional perturbation theory calculations were performed using the Phonon package of Quantum ESPRESSO. The dynamical matrix in the harmonic approximation was calculated at Γpoint using a linear response approach. The calculated IR spectrum was broadened by a Gaussian function with a FWHM of 10 cm^−1^.

## 4. Conclusions

Based on the Seebeck coefficient measurements and photo-electrochemical responses, we identified that the r-BS synthesized herein is a p-type semiconductor. Moreover, the distinct Fano resonance observed in the FT-IR results, which was ascribed to the Fano resonance between the E(2) (TO) phonon mode and electrons in the band structures of r-BS, indicates that the p-type carrier was intrinsically doped in the synthesized r-BS.

## Data Availability

Data is available on request from the corresponding author.

## Supplementary Materials

The following supporting information can be downloaded at: https://www.mdpi.com/article/10.3390/molecules28041896/s1, Figure S1: ESR measurement results of the r-BS sample before and after heating to 573 K; Figure S2: XRD pattern of the r-BS sample after Seebeck coefficient measurements (heating at 573 K); Figure S3: FT-IR spectra before and after Seebeck coefficient measurements (heating at 573 K); Figure S4: All Seebeck coefficient measurements for the r-BS sample; Figure S5: Thermogravimetric analysis (TGA) results of r-BS; Figure S6: Water desorption from r-BS during heating.

## Sample Availability

Samples of the compounds are available from the authors.
