# Supplementary material for: Rhombohedral Boron Monosulfide as a p-Type Semiconductor
Source: Molecules. 2023 Feb 16;28(4):1896. doi: 10.3390/molecules28041896 (PMC9963494; doi:10.3390/molecules28041896)
Supplement: Supplementary file 1 [file molecules-28-01896-s001.zip › molecules-2187262-supplementary.pdf]

molecules

Supplementary Information:

Rhombohedral Boron Monosulfide as a p-Type Semiconductor

Norinobu Watanabe [1], Keisuke Miyazaki [2], Masayuki Toyoda [3], Kotaro Takeyasu [4,5], Naohito Tsujii [6], Haruki Kusaka [1], Akiyasu Yamamoto [7], Susumu Saito [3], Masashi Miyakawa [8], Takashi Taniguchi [6], Takashi Aizawa [6], Takao Mori [6], Masahiro Miyauchi [2,*] and Takahiro Kondo [4,5,9,*]

[1] Graduate School of Pure and Applied Sciences, University of Tsukuba, Tsukuba 305-8573, Japan
[2] Department of Materials Science and Engineering, School of Materials and Chemical Technology, Tokyo Institute of Technology, Meguro-ku, Tokyo 152-8552, Japan
[3] Department of Physics, Tokyo Institute of Technology, Meguro-ku, Tokyo 152-8551, Japan
[4] Department of Materials Science and Tsukuba Research Center for Energy Materials Science, Faculty of Pure and Applied Sciences, University of Tsukuba, Tsukuba 305-8573, Japan
[5] R&D Center for Zero CO_2 Emission with Functional Materials, University of Tsukuba, Tsukuba 305-8573, Japan
[6] International Center for Materials Nanoarchitectonics (WPI-MANA), National Institute for Materials Science (NIMS), 1-1 Namiki, Tsukuba, Ibaraki 305-0044, Japan
[7] Institute of Engineering, Tokyo University of Agriculture and Technology, Tokyo 1838538, Japan
[8] Research Center for Functional Materials, National Institute for Materials Science, Tsukuba 305-0044, Japan
[9] Advanced Research Center for Quantum Physics and Nanoscience, Tokyo Institute of Technology, Meguro-ku, Tokyo 152-8551, Japan
* Correspondence: mmiyauchi@ceram.titech.ac.jp (M.M.); takahiro@ims.tsukuba.ac.jp (T.K.)

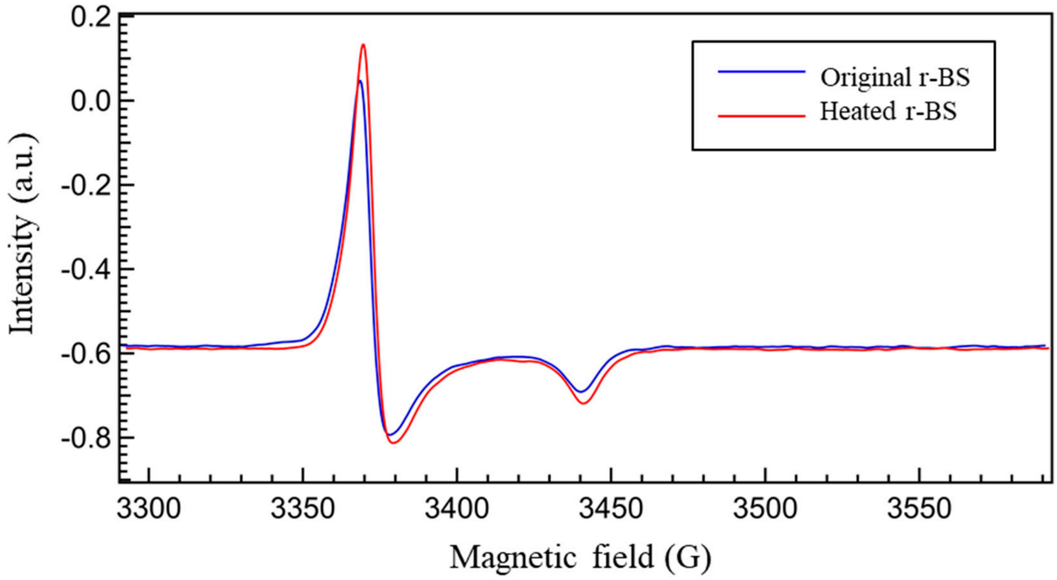

Figure S1. ESR measurement results of the r-BS sample before and after heating to 573K. The distinct peak indicates that r-BS has intrinsic defects. A negligible increase in the ESR signal after heating indicates that the r-BS defect amount does not change considerably after heating.

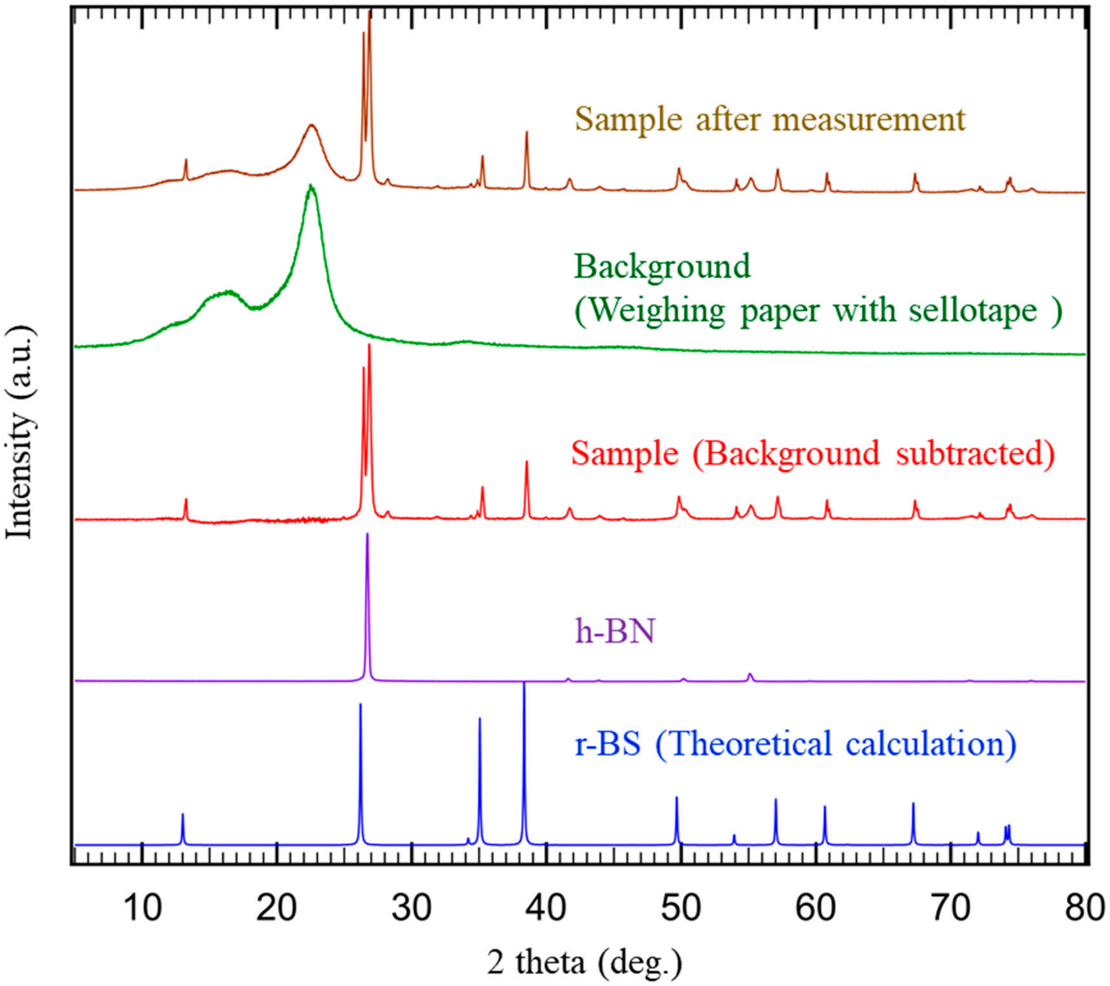

Figure S2. XRD pattern of the r-BS sample after Seebeck coefficient measurements (heating at 573 K). Because the XRD measurement was conducted with the sample on a weighing paper with adhesive tape, a corresponding overlapping signal was observed. This background signal was measured, and the subtracted XRD pattern was then derived. The results show that the sample used for the Seebeck coefficient (after heating at 573 K) measurement did not change after heating. Note that the appearance of h-BN peak is due to the inclusion of h-BN into the sample during the separation process of the capsule composed of h-BN.

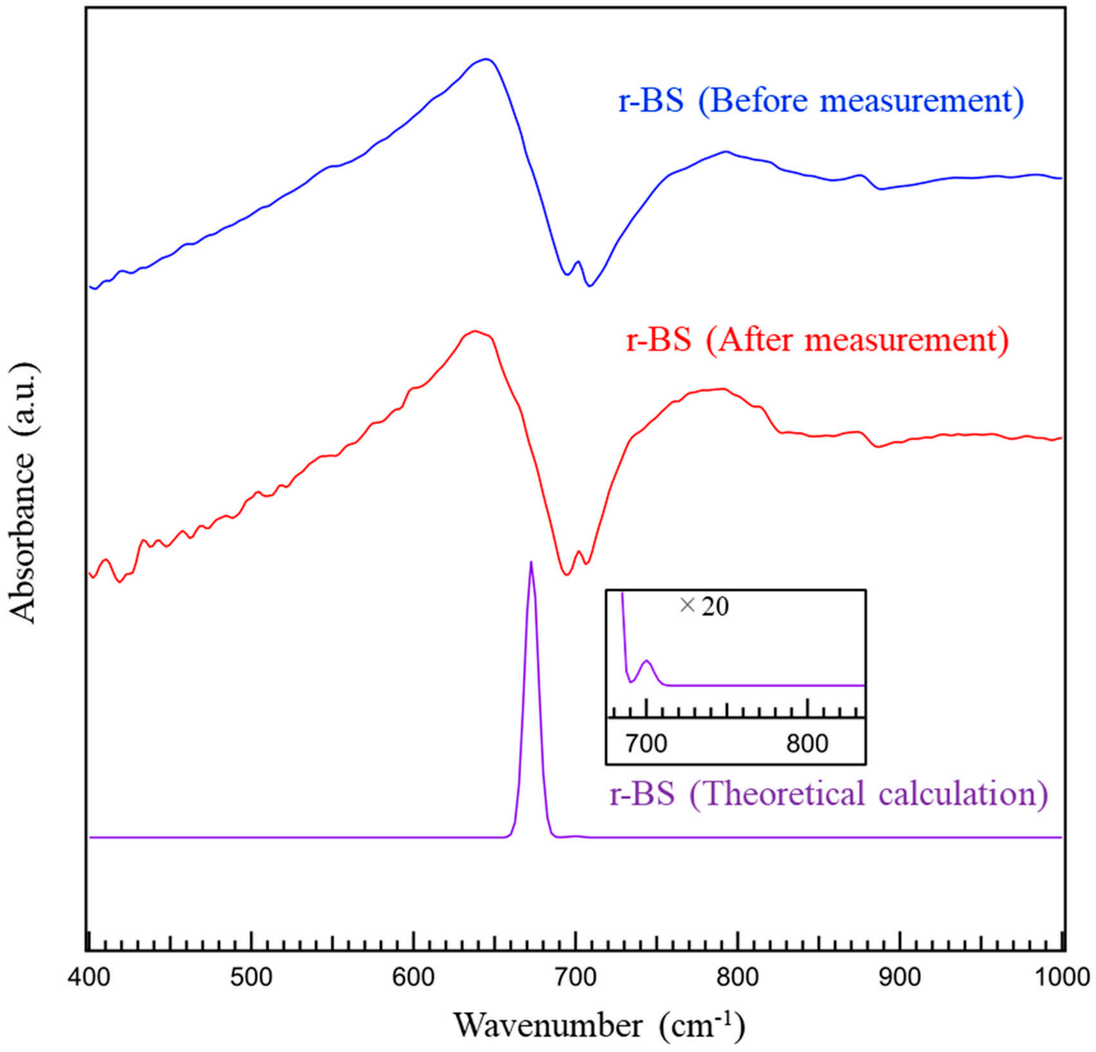

Figure S3. FT-IR spectra before and after the Seebeck coefficient measurements (heating at 573 K). Both are in close agreement, including the slight peak of the A1(2) (TO) vibrational mode. This result suggests that r-BS structure and properties are kept without changing during the Seebeck coefficient measurement process.

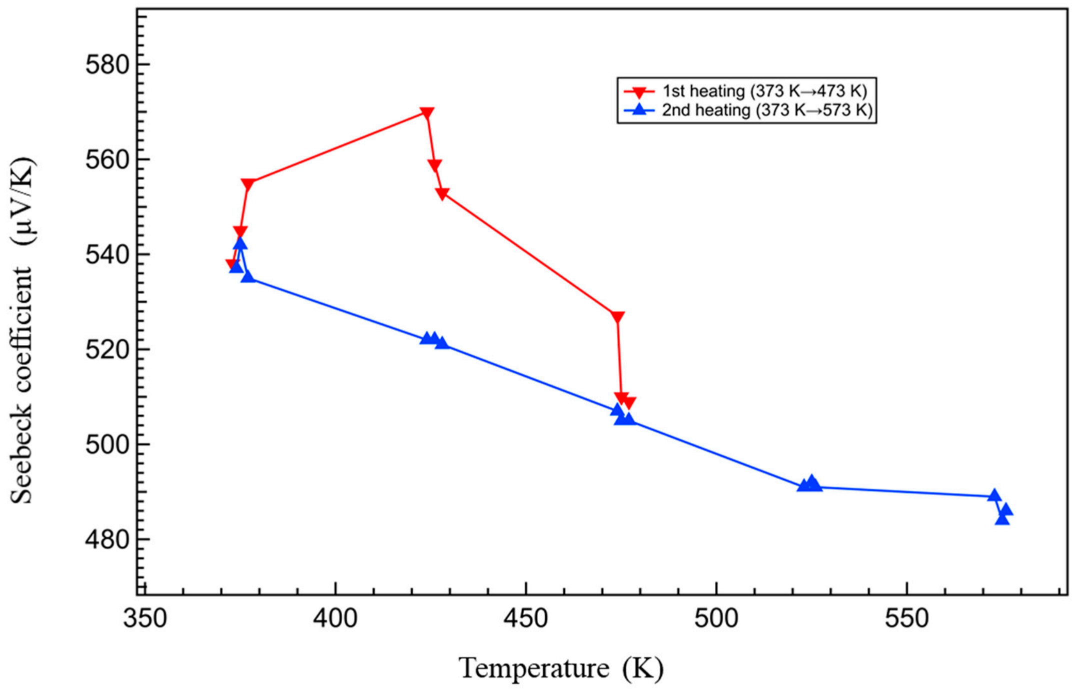

Figure S4. All Seebeck coefficient measurements for the r-BS sample conducted in this work. During the measurement, the heating program was set in the order of 373 K→473 K→373 K→573 K. The first heating program (373 K→473 K) is designated "1st heating" and the next program (373 K→573 K) is designated "2nd heating". In the 1st heating, the Seebeck coefficient increased when the temperature increased from 373 K to 420 K and thereafter decreased when the temperature further increased until 573 K. In contrast, the Seebeck coefficient decreased with an increase in the temperature in the 2nd heating. The decrease in Seebeck coefficient in the 2nd heating can be understood as an increase in the carrier concentration with increasing temperature. To clarify the behavior during the 1st heating, thermogravimetric analysis (TGA) (Figure S5), ESR (Figure S1), and gas analysis (Figure S6) were performed. TGA revealed that a weight loss of nearly 1% occurred in the range from 350 K to 450 K. Gas analysis indicated that the weight loss originated from the desorption of water adsorbed on r-BS. The ESR results before and after heating by TGA showed only a slight change with heating. Therefore, it was concluded that the weight loss confirmed by TGA was due to the desorption of water adsorbed on the sample in air. Moreover, the contact between the r-BS surface and the electrode improved upon the desorption of water in the 1st heating, and the Seebeck coefficient was accurately measured in the 2nd heating.

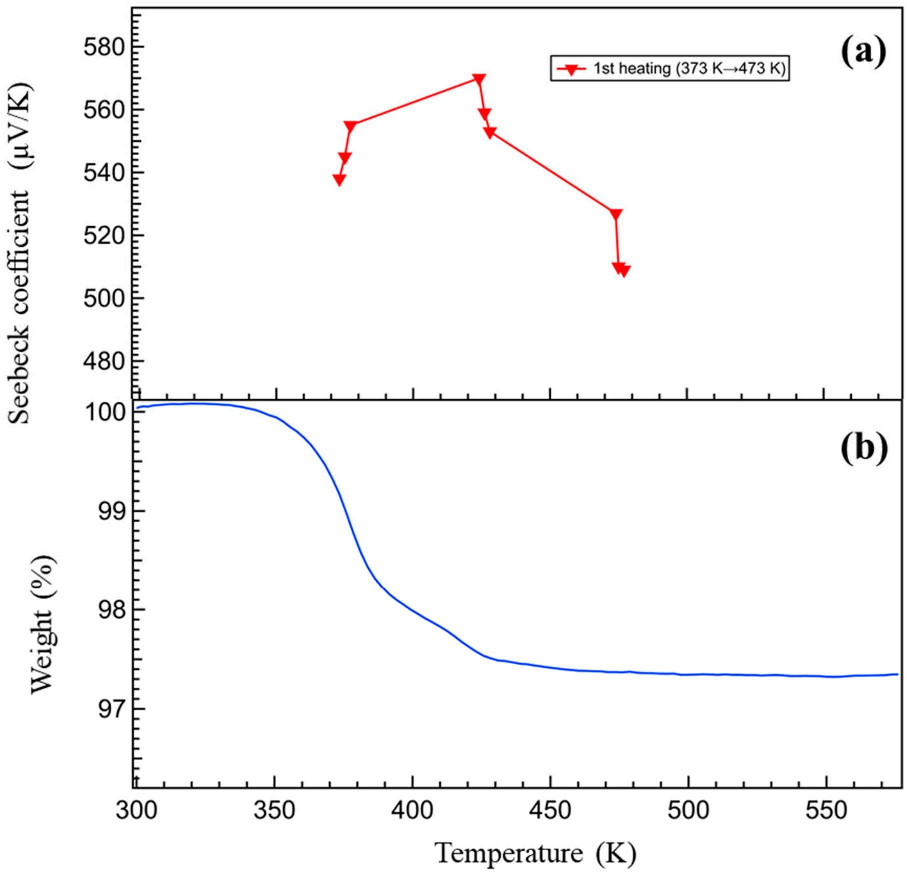

Figure S5. TGA of r-BS. The corresponding Seebeck coefficient measurement result during the 1st heating is shown. A weight loss of nearly 3% occurred in the range from 350 K to 450 K. Gas analysis (Figure S6) indicated that the weight loss originated from the desorption of water adsorbed on r-BS.

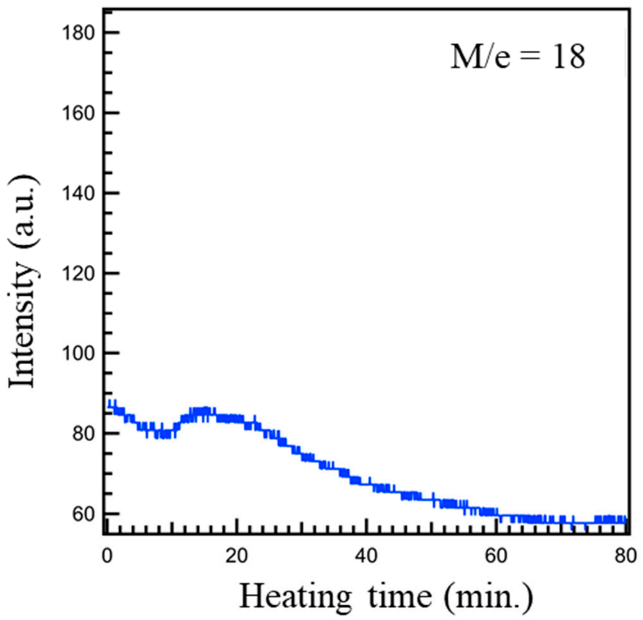

Figure S6. Water desorption from r-BS during heating. During the TGA analysis shown in Figure S5, mass number (M/e) 18 species (i.e., water) included in the exhausted gas in the Ar flow at the TGA system was monitored using the attached quadrupole mass spectrometer. Because time was not precisely matched with the temperature, the result is shown as a function of the heating time. The distinct increase in the intensity of water at around 20 min indicates that water desorbed from r-BS during the 1st heating.
